# Sudden Collapse in a Child Revealing a Malignant Coronary Anomaly: A Case of Congenital Anomaly of Left Coronary Artery Origin

**DOI:** 10.7759/cureus.84270

**Published:** 2025-05-17

**Authors:** Alisha Imran, Harry Carter, Jasmine Mensah, Mohammed Ahmed, Jhiamluka Solano

**Affiliations:** 1 Cardiology, Northern Lincolnshire and Goole NHS Foundation Trust, Grimsby, GBR; 2 Paediatrics, Northern Lincolnshire and Goole NHS Foundation Trust, Grimsby, GBR; 3 Resident Doctor Committee, Royal College of Physicians, London, GBR; 4 Education Committee, Academy of Medical Educators, Cardiff, GBR; 5 Cardiology, Scunthorpe General Hospital, Scunthorpe, GBR

**Keywords:** anomalous coronary artery, left anterior descending artery origin from rca, left coronary artery from the right sinus of the valsalva, unexplained syncope, unroof coronary sinus, unusal causes of persistent chest pain

## Abstract

Chest pain in children can be a common presentation with causes including idiopathic, musculoskeletal and respiratory conditions, but is rarely cardiac in origin. However, in rare cases, it may indicate life-threatening conditions such as anomalous origin of coronary arteries. Among these, anomalous origin of the left coronary artery from the right sinus (ALCRSV) with an intramural and inter-arterial course is particularly concerning due to its association with myocardial ischemia and sudden cardiac death. We present the case of a previously healthy seven-year-old girl who experienced an episode of chest pain and syncope, initially misattributed to heat syncope. On her second presentation, she was acutely unwell with signs of central cyanosis and ischemic changes on ECG. Further investigation with echocardiography revealed a suspected coronary anomaly. She was transferred to a specialist centre where a diagnosis of the left main coronary artery arising from the right sinus of Valsalva was confirmed. The patient underwent successful surgical correction, including coronary unroofing and reimplantation, and was started on spironolactone and aspirin. Her recovery was uneventful, and she was discharged with a scheduled follow-up. ALCRSV is a rare but serious congenital coronary anomaly that can present with exertional chest pain and syncope in children. Diagnosis is often challenging due to nonspecific symptoms and inconclusive initial investigations. However, timely recognition and surgical intervention can prevent fatal outcomes. This case emphasises the importance of maintaining a high index of suspicion for coronary anomalies in paediatric patients presenting with exertional syncope or ischemic ECG changes, even in the absence of prior cardiac history.

## Introduction

Congenital anomalies of the coronary arteries are uncommon, with a prevalence of approximately 1.3% among patients undergoing coronary angiography with no documented paediatric-specific incidence [1]. Among these, the anomalous origin of the left main coronary artery (LMCA) from the right sinus of Valsalva (RSV) is particularly significant due to its potential association with myocardial ischemia and sudden cardiac death, especially in young individuals and athletes [2,3]. This high-risk variant often follows an interarterial course between the aorta and pulmonary artery, where dynamic compression during physical exertion may precipitate arrhythmias or cardiac arrest [2,3]. Although many patients remain asymptomatic or present with nonspecific symptoms, increasing use of advanced imaging modalities has improved recognition and risk stratification [3-5]. Early diagnosis is critical, as timely surgical intervention may be life-saving in selected cases [6]. Here, we present the case of a paediatric patient with an anomalous LMCA arising from the RSV, with an initial intramural course within the ascending aortic wall, a variant associated with increased clinical risk.

## Case presentation

A seven-year-old girl, previously healthy with no significant past medical history, allergies, or family history, presented with recurrent episodes of chest pain and syncope. The first episode occurred during a physical education class. While running, she complained of chest pain and subsequently became intermittently unconscious. She was found on her hands and knees and was noted to be pyrexial, though she did not experience incontinence, vomiting, or other alarming symptoms. The patient was more alert by the time the ambulance arrived, and an ECG performed on-site showed no abnormalities. The serum blood glucose level and physical observations were normal, and she was discharged from the emergency department with a diagnosis of suspected heat syncope.

**Figure 1:**
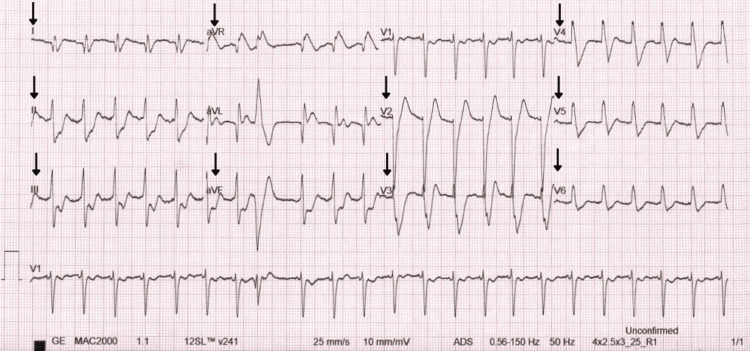
Admission 12-lead ECG showing ST segment depression widespread and ST segment elevation in aVR and precordial leads. ST elevation in leads avR and V2; ST depressions in leads I, II, III, aVF, V3-5

**Figure 2:**
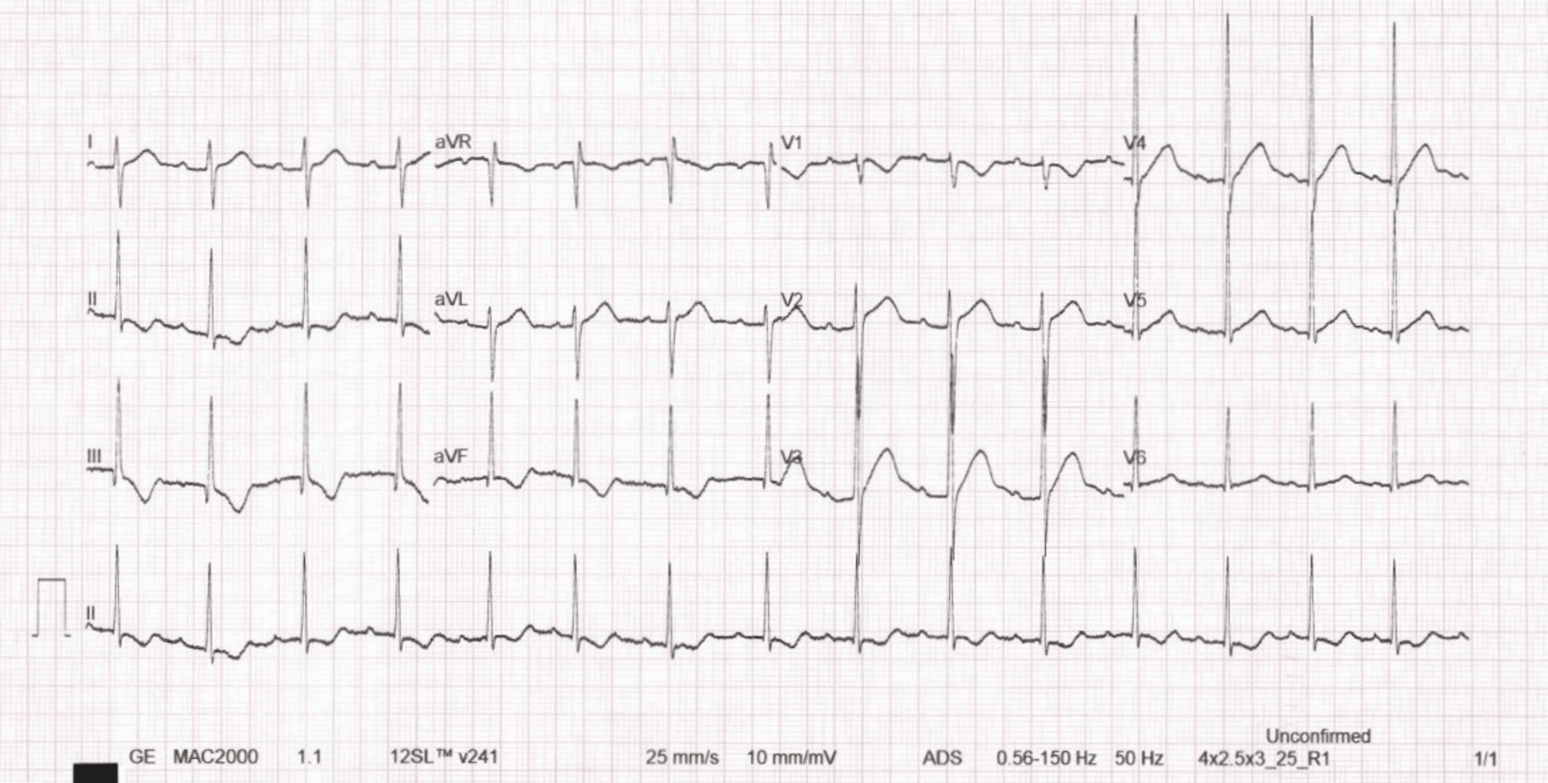
Post-initial assessment 12-lead ECG showing improved changes with wide T wave inversion in the inferior leads (I, II, and aVF).

A subsequent, more severe episode occurred after the patient ran home from the shops. She became dizzy and disoriented, complaining of chest pain, before collapsing at home. Upon arrival at the emergency department, the patient appeared grey, floppy, and was centrally cyanosed. She was immediately taken to resuscitation, given high-flow oxygen, a stat dose of broad-spectrum antibiotics, and resuscitation fluids. A 12-lead ECG revealed tachycardia at 131 bpm with concerning ischemic features, including marked ST elevation and depression in some leads (see Figure 1). A repeat ECG approximately 10 minutes later showed resolution of these changes (see Figure 2). A CT head scan was performed and was unremarkable. Chest X-ray suggested pulmonary venous congestion, particularly in the upper lobes. Blood cultures were negative.

Laboratory results revealed mild electrolyte abnormalities, a slightly elevated white blood cell count, and normal serum troponin levels, indicating no myocardial injury (see Table 1). A transthoracic echocardiogram performed in the emergency department initially showed no abnormalities, and hypertrophic obstructive cardiomyopathy was ruled out. However, a subsequent echocardiogram on the ward raised suspicion for an anomalous coronary artery arising from the right sinus. Additionally, it confirmed a normal ejection fraction with no valvulopathies (see Video 1).

**Table 1:** Admission and 2-hour post-admission blood test.

Parameter	On Arrival	2 hours Post-Arrival
pH (7.35 – 7.45)	7.258	7.326
Lactate (0.5-2 mmol/L)	3.9	1.5
Bicarbonate (22-26 mmol/L)	22.7	24.8
Base Excess (-2 to +2 mmol/L)	-4.6	-1.4
Haemoglobin (115-150 g/L)	114	109
Sodium (135-145 Na⁺, mmol/L)	143	144
Potassium (3.5-5.1 K⁺, mmol/L)	3.2	3.3

**Video 1:** Echocardiogram showing the left coronary artery arising from the right coronary sinus with an interarterial course using a parasternal short-axis view.

**Figure 3:**
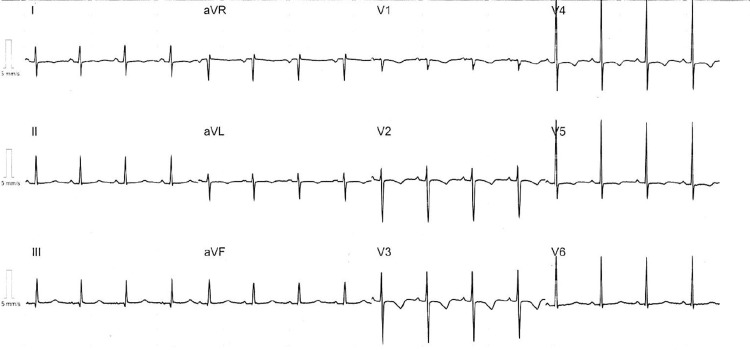
Post-surgical correction follow-up 12-lead ECG showing T wave inversion in the precordial leads.

The patient was transferred to a tertiary centre for further evaluation and management, where the diagnosis of anomalous origin of the left coronary artery (LCA) from the right sinus with an intramural and interarterial course was made by an echocardiogram at the congenital imaging department and this was later confirmed by CT coronary angiography (CTCA). The patient underwent surgery for this congenital abnormality, where, following median sternotomy, the LCA was deroofed and then re-implanted into the left coronary sinus. There were no reported intra/post-operative issues, and satisfactory flow through the re-implanted vessel was confirmed with a post-operative echocardiogram. An implantable loop recorder was later inserted for further monitoring, which demonstrated a consistent sinus rhythm prior to a successful discharge with oral aspirin for three months, spironolactone and furosemide to prevent post-operative complications, and a borderline low normal ejection fraction was noted in the follow-up echocardiogram. On follow-up, the patient remains stable, and a 12-ECG showed persistent T wave inversion in the precordial leads (see Figure 3). 

## Discussion

Congenital anomalies of coronary artery origin are rare, with the most clinically significant variant being the LMCA arising anomalously from the RSV with an initial intramural course within the ascending aortic wall [1]. This was the precise anomaly identified in our patient. It is associated with myocardial ischaemia and sudden cardiac death [2]. This risk stems from the dynamic compression of the LMCA between the aorta and pulmonary artery during periods of increased cardiac output, resulting in transient stenosis and subsequent ischaemia [7]. However, the underlying pathophysiology remains complex. These include a slit-like ostium, acute take-off angle, and compression of the intramural segment between the aorta and pulmonary artery during systole, particularly under increased haemodynamic demand (e.g., exercise). Most patients remain asymptomatic, although some may present with ischaemia-related symptoms such as angina pectoris, exertional chest pain, shortness of breath, dizziness, and syncope [8].

Diagnosing an anomalous left coronary artery from the right sinus of Valsalva (ALCRSV) is particularly challenging, as the majority of patients are asymptomatic [2]. These anomalies often result in inadequate myocardial perfusion, leading to intermittent hypoxia [8]. In symptomatic individuals, non-invasive tests such as ECG and echocardiography may still fail to detect underlying coronary artery disease or anomaly [9]. Absolute indications for surgical intervention include an anomalous origin of a coronary artery accompanied by a history of chest pain or syncope [10]. In our case, initial suspicion arose from an ECG showing tachycardia with ischaemic changes, notably transient ST elevation in lead aVR and widespread ST depression, an electrocardiographic pattern suggestive of global subendocardial ischaemia and possible left main coronary involvement. This, combined with exertional syncope, prompted an echocardiogram that revealed abnormal coronary anatomy [2]. This underscores the rarity of the condition in children and highlights the critical role of advanced imaging modalities, such as echocardiography, CTCA, and cardiac magnetic resonance imaging in facilitating early diagnosis and risk stratification [3].

ALCRSV is frequently diagnosed incidentally, as routine investigations often yield normal results [3]. Our patient had presented a year prior with a similar episode of syncope, but as her ECG was unremarkable, no further investigations were pursued. On her second presentation, the ECG abnormalities warranted further evaluation, including echocardiography. In children, chest pain and syncope are common and are most often benign. Typical causes include idiopathic, musculoskeletal, or respiratory conditions [11,12]. However, a more detailed evaluation is crucial in children presenting with abnormal physical findings or ECG changes, particularly when benign causes cannot explain symptoms such as exertional chest pain or syncope. These features can be indicative of underlying cardiovascular pathology, as was the case in our patient.

Surgical correction remains the definitive treatment for anomalous coronary artery origins [13,14]. Available surgical options include coronary reimplantation, coronary artery bypass grafting, unroofing, and patch ostioplasty [10]. According to the Congenital Heart Surgeons Society, unroofing is the most commonly performed procedure, accounting for 87% of cases. Our patient underwent unroofing, in addition to coronary artery translocation and reimplantation [6]. The goal of these procedures is to restore a normal anatomical course and connection of the anomalous coronary artery with the aorta. Outcomes following unroofing are excellent, with no reported operative mortality [15], and this was reflected in our patient’s uneventful post-operative course.

Post-operative antiplatelet therapy is crucial in reducing the risk of thrombus formation within the coronary arteries [15]. Furthermore, patients may remain at risk of heart failure following surgical correction, necessitating pharmacological support. Spironolactone is beneficial in this context as it improves cardiac function and mitigates fluid overload [12]. Accordingly, our patient was discharged on aspirin and spironolactone to prevent post-operative complications and a borderline low normal ejection fraction was noted in the follow-up echocardiogram. Pulmonary hypertension may develop after surgery, secondary to left ventricular diastolic dysfunction. Thus, diuretic therapy is often initiated pre-emptively [16]. The severity of post-operative symptoms is closely linked to the extent of left ventricular impairment, which, if significant, can result in symptoms of congestive heart failure, highlighting the importance of appropriate medical management.

## Conclusions

Anomalous origin of the LMCA from the RSV is a rare but potentially fatal congenital condition, particularly when associated with an intramural course. Although often asymptomatic, this anomaly can present with exertional syncope, chest pain, or even sudden cardiac death, particularly in young individuals. Early recognition remains challenging due to the often non-specific nature of symptoms and the limitations of initial non-invasive investigations. Our case highlights the importance of maintaining a high index of suspicion in patients with exertional symptoms and the utility of advanced imaging techniques for definitive diagnosis. Prompt surgical intervention, particularly coronary unroofing and reimplantation, can lead to excellent outcomes when performed in a timely manner. Post-operative pharmacological management plays a critical role in preventing complications and supporting myocardial recovery. Clinicians should consider anomalous coronary anatomy in the differential diagnosis of unexplained syncope or exertional chest pain, especially in younger patients or those with recurrent symptoms.
